# Study of prostate-specific antigen levels during salvage radiotherapy after prostate cancer surgery

**DOI:** 10.1186/s12894-023-01323-5

**Published:** 2023-10-04

**Authors:** Takuya Aizawa, Toshiya Maebayashi, Naoya Ishibashi, Masakuni Sakaguchi, Akahiko Sato, Kenya Yamaguchi

**Affiliations:** 1 Department of Radiology, Nihon University School of Medicine, Itabashi-ku, Tokyo, 173-8610 Japan; 2 Department of Radiology, Nihon University Hospital, Chiyoda-ku, Tokyo, 101- 8309 Japan; 3 Department of Urology, Nihon University Hospital, Chiyoda-ku, Tokyo, 101-8309 Japan

**Keywords:** PSA, PSADT, Prostate cancer, Radiotherapy, Biochemical recurrence

## Abstract

**Background:**

Administration of adjuvant or salvage radiotherapy (RT) after prostate cancer (PCa) surgery is supported by clinical evidence and is a widely adopted strategy. On occasion, we detect changes in prostate-specific antigen (PSA) levels, such as a transient elevation or decline, during RT. Thus, we retrospectively investigated the frequency of changes in PSA levels, their associations with histopathological parameters, PSA doubling time (PSADT), and biochemical recurrence (BR) of PCa.

**Methods:**

This study included 23 consecutive patients who underwent surgery for PCa between 2012 and 2019, received salvage RT without hormone therapy, and exhibited changes in PSA levels during RT. The prostatic bed was irradiated with a total dose of 64 to 66 Gy. BR was defined as consecutive PSA levels exceeding 0.2 ng/mL or having to start hormone therapy because of PSA elevation after salvage RT.

**Results:**

During salvage RT after PCa surgery, PSA levels transiently increased in 11 patients (47.8%) and decreased in 12 (52.2%). When factors associated with BR were examined in patients with transient PSA elevation, seminal vesicle invasion and preoperative PSA values were identified as being statistically significant. When factors for BR were examined in patients with a decline in PSA levels, the Gleason score and PSADT were identified as being significant. Among the cases of a decline in PSA levels during salvage RT, those who received a radiation dose of less than 36 Gy did not experience BR. Similarly, patients who exhibited changes in PSA levels during salvage RT and did not have perineural invasion did not experience BR.

**Conclusion:**

This is the first study to examine the histopathological factors possibly affecting BR in patients undergoing salvage RT after PCa surgery. The results indicate that in patients with transient PSA elevation, seminal vesicle invasion is a significant risk factor. On the other hand, in patients with a decline in PSA levels during irradiation, the Gleason score and perineural invasion were found to be potential risk factors for BR. These findings suggest that a thorough examination of postoperative histopathological results may be necessary for the optimal management of patients with PCa.

## Background

Administration of adjuvant [1–3] or salvage [4] radiotherapy (RT)after prostate cancer surgery is supported by extensive clinical evidence and is a widely adopted strategy. There have been 9 studies examining changes in prostate-specific antigen (PSA) levels during salvage RT [5–13]. All but one of these studies, showed that such changes are factors possibly associated with biochemical or clinical recurrence [5–8, 10–13], while the one study found that such changes were not linked to recurrence [9]. This study was thus designed to retrospectively examine patients with changes in PSA levels during RT after prostate cancer surgery, focusing on the detailed pathological findings.

## Methods

### Ethics statement

All procedures performed were in accordance with the ethical standards of the institutional and/or national research committee and with the 1964 Helsinki declaration and its later amendments or comparable ethical standards. This study was approved by the institutional review board of our institution, and informed consent was obtained from all participants. (IRB approval number RK-190611-3)

### Patient characteristics

The subjects were 75 patients who received irradiation after prostate cancer surgery between 2012 and 2019. Among them, we selected 23 consecutive patients without lymph node metastasis who received salvage RT without hormone therapy after prostate cancer surgery and exhibited changes in PSA levels during irradiation. The tests before irradiation confirmed the absence of metastasis or local recurrent masses in all patients. Salvage RT was indicated for patients who had not experienced biochemical recurrence (BR) after prostate cancer surgery and those whose postoperative PSA levels did not reach the measurement limit.

The follow-up periods ranged from 26 to 95 months (median: 45 months). The patient ages ranged from 54 to 76 years (median: 67 years). The postoperative tumor stage was T2c or higher in 87% of all patients and T3 in 47.8%. According to the D’Amico risk classification, 2 and 21 patients had intermediate risk and high risk, respectively. Table 1 shows histopathological findings, Gleason scores, PSA levels, and other relevant data.

**Table 1:** Patient characteristics

	All patients (n = 23)	Transient PSA elevation (n = 11)	PSA decline (n = 12)	p-value
Follow-up (months)				0.14
Median	63	44	57	
Range	30–112	30–112	53–95	
Age (years)				0.48
Median	67	67	67	
Range	54–76	57–76	54–73	
T-Stage				0.14
T2a/T2c	3/ 9	1/ 5	2/ 6	
T3a/T3b	7/ 4	5/ 2	2/ 2	
Initial PSA (ng/ml)				0.16
Median	11.37	13.83	8.72	
Range	3.22–40.6	3.22–40.6	4.76–17.96	
Gleason score				0.79
7	18	8	10	
8/ 9	3/ 2	3/ 1	0/ 1	
D’Amico risk group classification				
Low risk	0	0	0	
Intermediate risk	2	0	2	
High risk	21	11	10	
Pathological diagnosis				
ly1	11	7	4	0.16
pn1	20	9	11	0.51
sv1	4	2	2	0.93
v1	3	3	0	0.08
EPE1	12	6	6	0.84
RM1	17	9	8	0.43

**Abbreviations:** PSA: Prostate-Specific Antigen, ly1: lymphatic invasion, pn1: perineural invasion, sv1: seminal vesicle invasion, v1: vessel invasion, EPE: extraprostatic extension, RM: resection margin

### Statistical analysis

The differences in biochemical failure (BF) were expressed at a 5% significance level employing a two-tailed log-rank test. Age, initial PSA, PSA before RT ≤ 0.3, PSA nadir, PSA velocity (PSAV), PSA doubling time (PSADT), period from post-operation to RT ≤ 3 years, T-stage, Gleason Score ≤ 7, seminal vesicle invasion (SV1), lymphatic invasion (Ly1), vessel invasion (V1), perineural invasion (pn1), resection margin (RM1), extraprostatic extension (EPE1) and RT dose at PSA measurement were analyzed to identify factors predicting BF. All calculations and survival displays were conducted using the SPSS 21.0 J statistical software (SSPS Inc., Chicago, IL, USA). Acute and late toxicities were graded according to the National Cancer Institute-Common Terminology Criteria (NCI-CTC), Version4.0 [14].

### Definition of recurrence after salvage radiotherapy

Recurrence was defined as consecutive PSA levels exceeding 0.2 ng/mL or requiring the initiation of hormone therapy due to PSA elevation after RT.

### Radiation therapy

The clinical target volume (CTV) consisted of the site after prostatectomy, and the planning target volume (PTV) was set as the CTV with 8-mm margins. However, when a radiation field was set on the rectal surface, PTV was modified to consist of the CTV with approximately 5-mm margins. The prescribed dose was 64~66 Gy at the isocenter, administered as 2 Gy per fraction. RT was applied using a 10-MV X-ray Synergy (Elekta, Crawly, United Kingdom) and performed with 7 fixed ports. For the treatment planning, the Xio or Monaco (Elekta CMS Software, St. Louis, MO, USA) was used, and calculations were performed using the superposition technique. This point was set to ensure that the 95% isodose line would satisfy the PTV. For cases treated after 2015, Image-Guided Radiation Therapy was employed.

## Results

### Changes in PSA levels in all patients

**Fig. 1:**
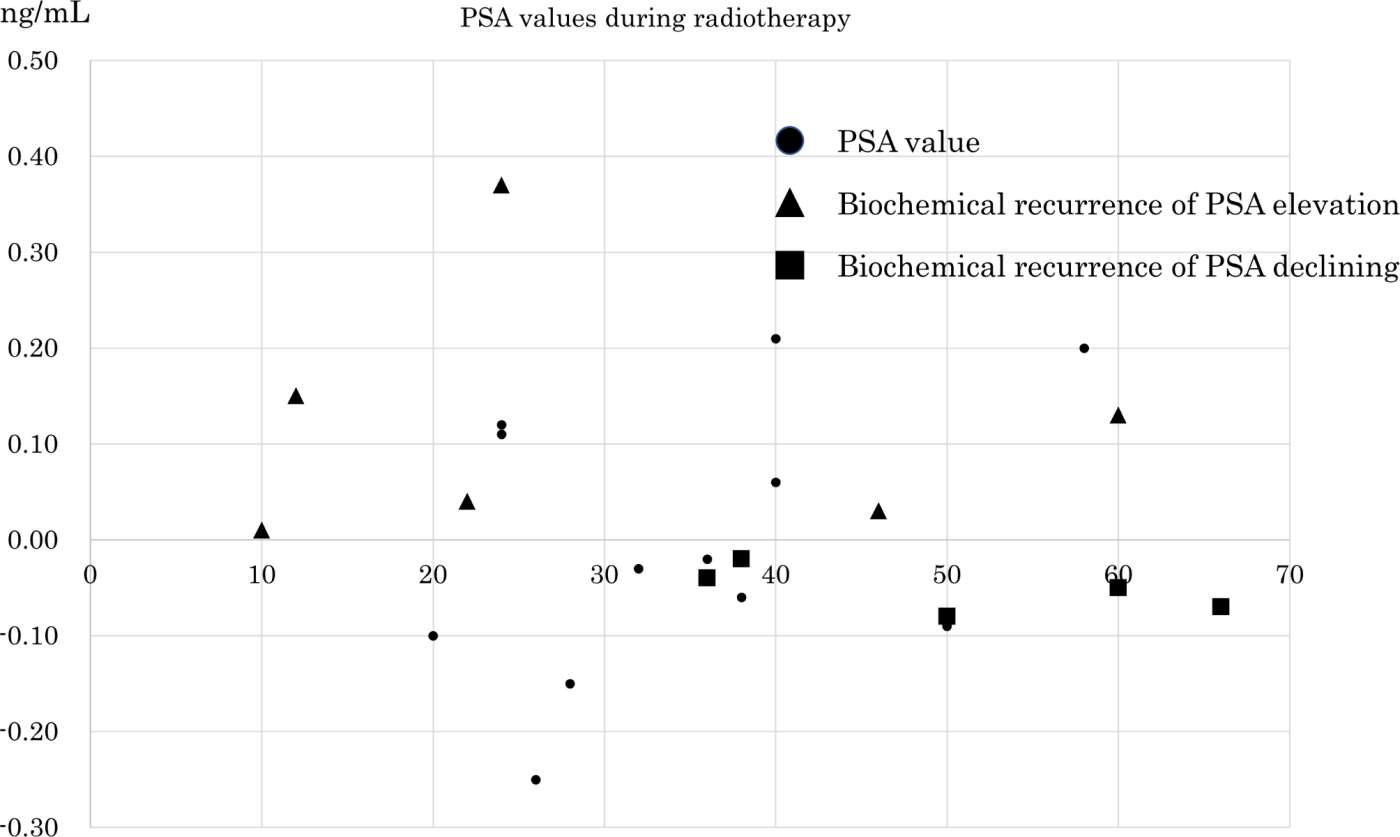
PSA levels during radiotherapy Among the cases with elevated PSA during irradiation, those with recurrent disease are denoted by black triangles, and among those with decreased PSA during irradiation, the patients with recurrent disease are denoted by black squares

During salvage RT, PSA levels transiently increased in 11 patients (47.8%) and decreased in 12 (52.2%). The radiation doses at the time of PSA measurement ranged from 10 to 60 Gy (median: 34 Gy) (Fig. 1). Before irradiation, the median PSA level was 0.32 ng/mL, the median PSADT was 129.1 days, and the median PSAV was 0.65 ng/ml/year. Recurrence after salvage RT was detected in 11 patients (45.8%), and the median time to recurrence was 28 months. The details are shown in Table 2.

**Table 2:** List of factors examined

	All patients (n = 23)	Transient PSA elevation (n = 11)	PSA decline (n = 12)	p-value
Recurrent cases	12 (52.2%)	6 (54.5%)	6 (50%)	0.837
Time to recurrence after salvage RT (months)				0.593
Median	26	21	26	
Range	2–55	7–55	2–29	
Period from post-operation to RT (months)				0.742
Median	14	11	18.5	
Range	3–65	4–65	3–44	
Pre-RT PSA (ng/ml)				0.089
Median	0.32	0.41	0.28	
Range	0.1–1.77	0.1–1.77	0.19–0.61	
PSADT (day)				0.037
Median	129.1	63.16	254.1	
Range	22.8–471.1	28.8–257	38.9–471.1	
PSAV (ng/ml/year)				0.071
Median	0.65	1.1	0.172	
Range	0.086–6.74	0.102–6.74	0.086–1.648	
Transient PSA elevation value (ng/ml)				
Median		0.12		
Range		0.01–0.37		
Decline in PSA value (ng/ml)				
Median			0.07	
Range			0.02–0.25	
Dose at PSA measurement (Gy)				0.281
Median	36	24	37	
Range	10–60	10–60	20–66	

**Abbreviations**: PSA: Prostate-Specific Antigen, RT: radiotherapy, PSADT: Prostate-Specific Antigen Doubling Time, PSAV: *prostate*-specific antigen velocity, Gy: Gray

### Transient PSA elevation during irradiation

In the 11 patients with transient PSA elevation, the median radiation dose was 24 Gy at the time of PSA measurement during salvage RT. Before irradiation, the median PSA level was 0.41 ng/mL, the median PSADT was 63.16 days, and the median PSAV was 1.1 ng/ml/year. BR after salvage RT was detected in 6 patients (54.5%), and the median time to recurrence was 21 months (Table 2). In 2 of the patients with recurrence, PSA levels decreased after rising during irradiation. However, approximately 2 months later, both patients exhibited increases in their PSA levels. In the other 4 patients, PSA levels decreased after transient elevation and increased again 28 to 55 months later (median 34.5 months). To date, no BR has been detected in the other 5 patients (Fig. 1).

### Decline in PSA levels during irradiation

In the 12 patients with a decline in PSA levels, the median radiation dose was 37 Gy at the time of PSA measurement during salvage RT. Before irradiation, the median PSA level was 0.28 ng/mL, the median PSADT was 254.1 days, and the median PSAV was 0.172 ng/ml/year. BR after irradiation was detected in 6 patients (41.7%), and the median time to recurrence was 26 months (Table 2). To date, no BR has been detected in the other 6 patients (Fig. 1).

### Association between biochemical recurrence and changes in PSA levels during salvage radiotherapy

**Fig. 2:**
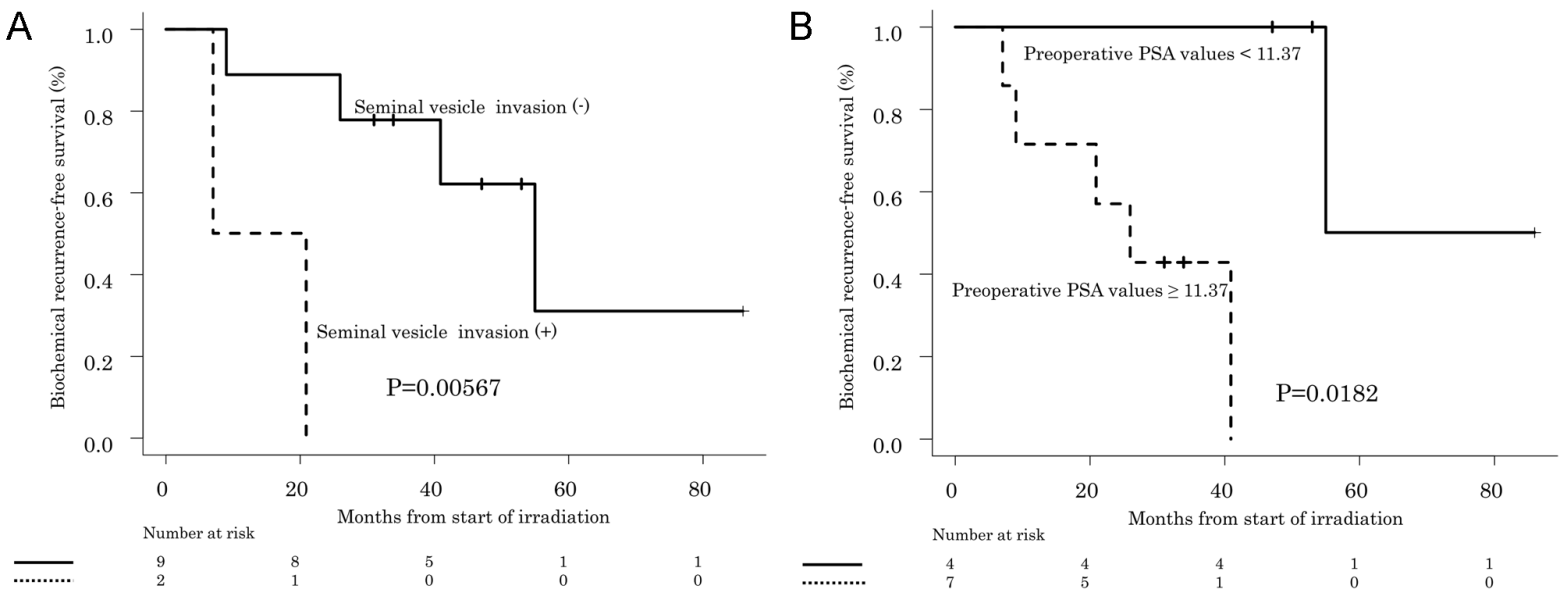
** A**: Biochemical recurrence-free survival of cases with and without seminal vesicle invasion among those with PSA elevation PSA. *P* values were calculated by the log-rank test, with stratification according to the radiation therapy group **B**: Biochemical recurrence-free survival and preoperative PSA in cases with PSA elevation. *P* values were calculated by the log-rank test, with stratification according to the radiation therapy group

**Fig. 3:**
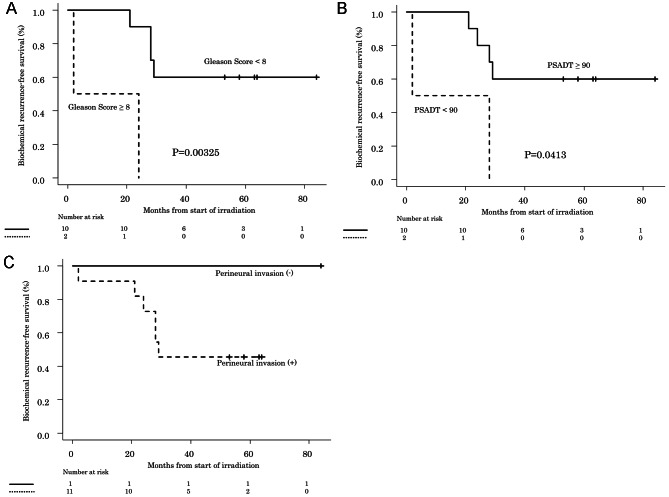
** A**: Biochemical recurrence-free survival and Gleason Score ≧ 8 or < 7 in cases with a decline in PSA levels. *P* values were calculated by the log-rank test, with stratification according to the radiation therapy group. **B**: Biochemical recurrence-free survival and PSADT ≧ 90 or < 90 (day) in cases with a decline in PSA levels. *P* values were calculated by the log-rank test, with stratification according to the radiation therapy group. **C**: Biochemical recurrence-free survival of cases with and without neural invasion among cases with a decline in PSA levels. *P* values were calculated by the log-rank test, with stratification according to the radiation therapy group

When factors possibly associated with BR after salvage RT were examined, seminal vesicle invasion (Fig. 2A) and preoperative PSA values (Fig. 2B) were identified as being significantly associated with transiently elevated PSA during irradiation (Table 3), while the Gleason Score (Fig. 3A) and PSADT (Fig. 3B) were identified as significant factors in patients with a decline in PSA levels during irradiation (Table 3).

**Table 3:** Univariate analysis of biochemical failure in cases with a decline in PSA levels or PSA elevation

Factor	P- value of biochemical recurrence with PSA decline	P-value of biochemical recurrence with PSA elevation
Age	0.123	0.415
Initial PSA	0.656	**0.0385**
PSA before RT ≤ 0.3	0.964	0.153
PSA nadir Yes/No	0.191	0.155
PSAV	0.109	0.992
PSADT ≥ 90 or ≺90	**0.0413**	0.581
Period from post-operation to RT ≤ 3 years	0.408	0.544 (Only one case)
Gleason Score ≤ 7 or > 8	**0.00325**	0.219
SV1	0.101	**0.00567**
Ly1	0.302	0.737
V1	None (No V1 cases)	0.167
pn1	0.386 (Only one pn(-) case)	0.879
RM1	0.302	0.453
EPE1	0.849	0.123

**Abbreviations:** RT: radiotherapy, PSA: Prostate-Specific Antigen, PSADT: Prostate-Specific Antigen Doubling Time, PSAV: prostate-specific antigen velocity, Ly1: lymphatic invasion, pn1: perineural invasion, SV1: seminal vesicle invasion, V1: vessel invasion, EPE1: extraprostatic extension, RM1: resection margin

Among patients with a decline in PSA levels during irradiation, those receiving a radiation dose of less than 36 Gy after initiation of irradiation (Fig. 1), those with a PSA level decrease of 0.1 or more (Fig. 1) and those without perineural invasion (Fig. 3C) did not experience BR. Table 3 shows the results of analyzing factors for BR after salvage RT in patients with changes in PSA levels during irradiation.

### Adverse events

In terms of adverse events, the acute phase showed Grade 1 genitourinary toxicity (GU) in 65.2% of cases and Grade 1 gastrointestinal toxicity (GI) in 17.3%, with no occurrences of Grade 2 or higher adverse events. In the late phase, 3 patients (13%) experienced Grade 2 GU toxicity, including 2 cases of urinary incontinence and 1 case of urinary retention. All of these events occurred more than 3 years after radiotherapy. No GI adverse events were observed. Additionally, no Grade 3 or higher adverse events were reported.

## Discussion

After radical prostatectomy for localized prostate cancer, PSA levels increase in 25–30% of patients. If left untreated, two-thirds of these patients will likely develop metastatic lesions and die from prostate cancer [15, 16]. For this reason, administration of postoperative adjuvant [1–3] or salvage RT [4] is a widely adopted strategy. In addition, recent clinical studies have shown that a combination of salvage RT and hormone therapy is associated with improved outcomes [17–19].

Despite salvage RT being a widely adopted strategy, our literature search yielded only 9 articles [5–13] and one review [20] on changes in PSA levels during RT. All but one of these articles showed that such changes might be a factor impacting biochemical or clinical recurrence [5–8, 10–13], while the other article described changes in PSA levels as not being a factor associated with recurrence [9].

The results of our study examining patients with a decline in PSA levels during salvage RT were consistent with those of previously reported studies, showing that a decrease of 0.2 or more in PSA levels during irradiation affects BR [20]. However, our results contradicted those of previous studies showing BR to be affected by decreases in PSA levels after radiation doses reached and exceeded 45 Gy [5, 12, 13, 20]. In fact, our results revealed no evidence of BR in patients in whom PSA levels declined relatively early after the initiation of RT. The decline in PSA levels in the latter half of RT is speculated to be attributable to tumor lysis [5, 10]; in our study, however, only 2 patients exhibited a decrease in the PSA level of 0.15 ng/mL or more during the first half of RT. As shown in the nomogram reported by Gunnlaugsson et al., it is speculated that PSA levels decrease over time [10]. Moreover, our results are consistent with reports showing that the Gleason score and PSADT affects BR [10]. Further analysis of the detailed pathological findings suggests that there might be an association between changes in PSA levels and recurrence.

When we examined patients with transient PSA elevation during salvage RT, seminal vesicle invasion was identified as a factor associated with BR after salvage RT. Zhong et al. reported that seminal vesicle invasion was not a factor impacting BR after salvage RT [21], while Cardoso et al. reported that seminal vesicle invasion was such a factor in patients in whom PSA levels transiently increased or remained unchanged during irradiation [12]. Thus, a transient PSA elevation might be associated with pathological factors, such as seminal vesicle invasion. In addition, patients in whom PSA levels transiently increased relatively early after the initiation of RT experienced BR. These results indicate that the impact on PSADT may have resulted in PSA elevation, as reported by Lohm [9]. However, because the PSA elevation due to irradiation of normal prostate tissues reportedly peaks in 2 to 4 weeks [22, 23], it may be important to perform high-quality pretreatment tests that can determine the presence or absence of residual prostate tissues and other factors.

In this study, we detected no PSA recurrences in patients without perineural invasion whose PSA levels decreased during salvage RT. Because recent studies have shown perineural invasion to be associated with survival [24, 25], this type of tumor spread may be an important factor, unlike hematogenous and lymphatic invasion. On the other hand, Zhong et al. [21] reported that perineural invasion was not a factor associated with BR after salvage RT. Thus, further studies might be needed to determine the combined effects of changes in PSA levels during salvage RT and perineural invasion.

Although our follow-up period was long, the sample size was small in this study. Thus, possible biases should be taken into sufficient consideration. However, additional analysis of detailed pathological findings raised the possibility that changes in PSA levels during salvage RT might affect BR. If highly accurate tests, such as prostate-specific membrane antigen positron emission tomography/computed tomography [26], could be performed before salvage RT, small residual tumors might well be detectable. Consequently, it is anticipated that various questions will be answered by future research in this area. In addition, the results of a currently ongoing study (NCT04858880) are awaited [13].

## Conclusion

While there are reports of transient elevations or decreases in PSA levels during salvage RT after prostate cancer surgery, this study provides the first detailed examination of histopathological results. In patients with transient PSA elevation during irradiation, seminal vesicle invasion was found to be a factor affecting BR. In addition, the Gleason score, PSADT and perineural invasion may affect BR in patients with a decline in PSA levels during irradiation. In future studies, changes in PSA levels during salvage RT may need to be examined in combination with detailed pathological results.

## Data Availability

The data that support the findings of this study are available from the corresponding author, T.Maebayashi, upon reasonable request.

## Abbreviations

RT: radiotherapy
PSA: prostate-specific antigen
PSADT: PSA doubling time
BF: biochemical failure
PSAV: PSA velocity
SV1: seminal vesicle invasion
Ly1: lymphatic invasion
V1: vessel invasion
pn1: perineural invasion
RM1: resection margin
EPE1: extraprostatic extension
CTV: clinical target volume
PTV: planning target volume
